# The Role of the Vitamin D Receptor in the Pathogenesis, Prognosis, and Treatment of Cutaneous Melanoma

**DOI:** 10.3389/fonc.2021.743667

**Published:** 2021-10-06

**Authors:** Alyssa L. Becker, Evan L. Carpenter, Andrzej T. Slominski, Arup K. Indra

**Affiliations:** ^1^ Department of Pharmaceutical Sciences, College of Pharmacy, OSU, Corvallis, OR, United States; ^2^ John A. Burns School of Medicine at the University of Hawai’i at Mānoa, Honolulu, HI, United States; ^3^ Department of Dermatology, University of Alabama at Birmingham, Birmingham, AL, United States; ^4^ Cancer Chemoprevention Program, Comprehensive Cancer Center, University of Alabama at Birmingham, Birmingham, AL, United States; ^5^ Knight Cancer Institute, Oregon Health & Science University (OHSU), Portland, OR, United States; ^6^ Department of Biochemistry and Biophysics, Oregon State University (OSU), Corvallis, OR, United States; ^7^ Linus Pauling Science Center, Oregon State University (OSU), Corvallis, OR, United States; ^8^ Department of Dermatology, Oregon Health & Science University (OHSU), Portland, OR, United States

**Keywords:** melanoma, vitamin D receptor (VDR), vitamin D3 metabolite, therapy, pathogenesis, tumor microenvironment, polymorphisms, heterodimers

## Abstract

Melanoma is the malignant transformation of melanocytes and represents the most lethal form of skin cancer. While early-stage melanoma localized to the skin can be cured with surgical excision, metastatic melanoma often requires a multi-pronged approach and even then can exhibit treatment resistance. Understanding the molecular mechanisms involved in the pathogenesis of melanoma could lead to novel diagnostic, prognostic, and therapeutic strategies to ultimately decrease morbidity and mortality. One emerging candidate that may have value as both a prognostic marker and in a therapeutic context is the vitamin D receptor (VDR). VDR is a nuclear steroid hormone receptor activated by 1,25 dihydroxy-vitamin D3 [calcitriol, 1,25(OH)_2_D3]. While 1,25 dihydroxy-vitamin D3 is typically thought of in relation to calcium metabolism, it also plays an important role in cell proliferation, differentiation, programmed-cell death as well as photoprotection. This review discusses the role of VDR in the crosstalk between keratinocytes and melanocytes during melanomagenesis and summarizes the clinical data regarding VDR polymorphisms, VDR as a prognostic marker, and potential uses of vitamin D and its analogs as an adjuvant treatment for melanoma.

## Introduction

The worldwide incidence of melanoma has steadily increased over the past several decades with the annual incidence rising as rapidly as 4-6% in certain regions (1). In 2021, it is estimated that approximately 106,110 new melanomas will be diagnosed in the United States alone (2). While the incidence of melanoma is greatest in older adult populations, peaking at the sixth decade of life in the United States, it is also one of the most common malignancies found in adolescent and young adult populations (1, 3, 4). In addition to being a relatively ubiquitous cancer, melanoma is the most lethal skin cancer resulting in 9,008 deaths per year in the United States between the years of 2012-2016 (1).

Cutaneous melanoma results from the malignant transformation of predominantly melanocytes (5). Since these pigment producing cells are generally confined to the epidermis of the skin the appearance of vertical growth or Breslow thickness play key roles in determining the aggressiveness of the tumor and its likelihood of metastasis (6). For instance, a stage 0 melanoma is confined only to the epidermis and does not involve nearby dermis or spread to lymph nodes and distant organs. Whereas any melanoma that involves distant metastases is classified as a stage IV tumor.

In early-stage melanoma surgical excision is often curative when the tumor is localized to the skin (1). However, following progression to metastatic melanoma treatment becomes more complex and may include inhibition of metastasis, immunotherapy, targeted inhibition of the mitogen-activated protein kinase (MAPK) pathway, and/or radiation therapy (7, 8). Despite initial improvements these treatments are not fully effective and the cancer is terminal in many cases (9). Reversing this trend is the challenge ahead of melanoma investigators and clinicians, where a more thorough understanding of the molecular mechanisms involved in the pathogenesis of melanoma could lead to novel diagnostic, prognostic, and therapeutic strategies, ultimately resulting in a decreased mortality rate.

One emerging candidate for both targeted therapy and prediction of prognosis is the vitamin-D-receptor (VDR) (10–13). VDR is a nuclear steroid hormone receptor that is found in several organs, including the skin (14). VDR is activated by 1,25 dihydroxy-vitamin D3 (calcitriol, 1,25(OH)_2_D3) which, in addition of regulating body calcium metabolism, is involved in many pleiotropic activities including regulation of cell proliferation, differentiation, and programmed cell death as well as in photoprotection (12, 13, 15–21).

## Activation of Vitamin D

**Figure 1:**
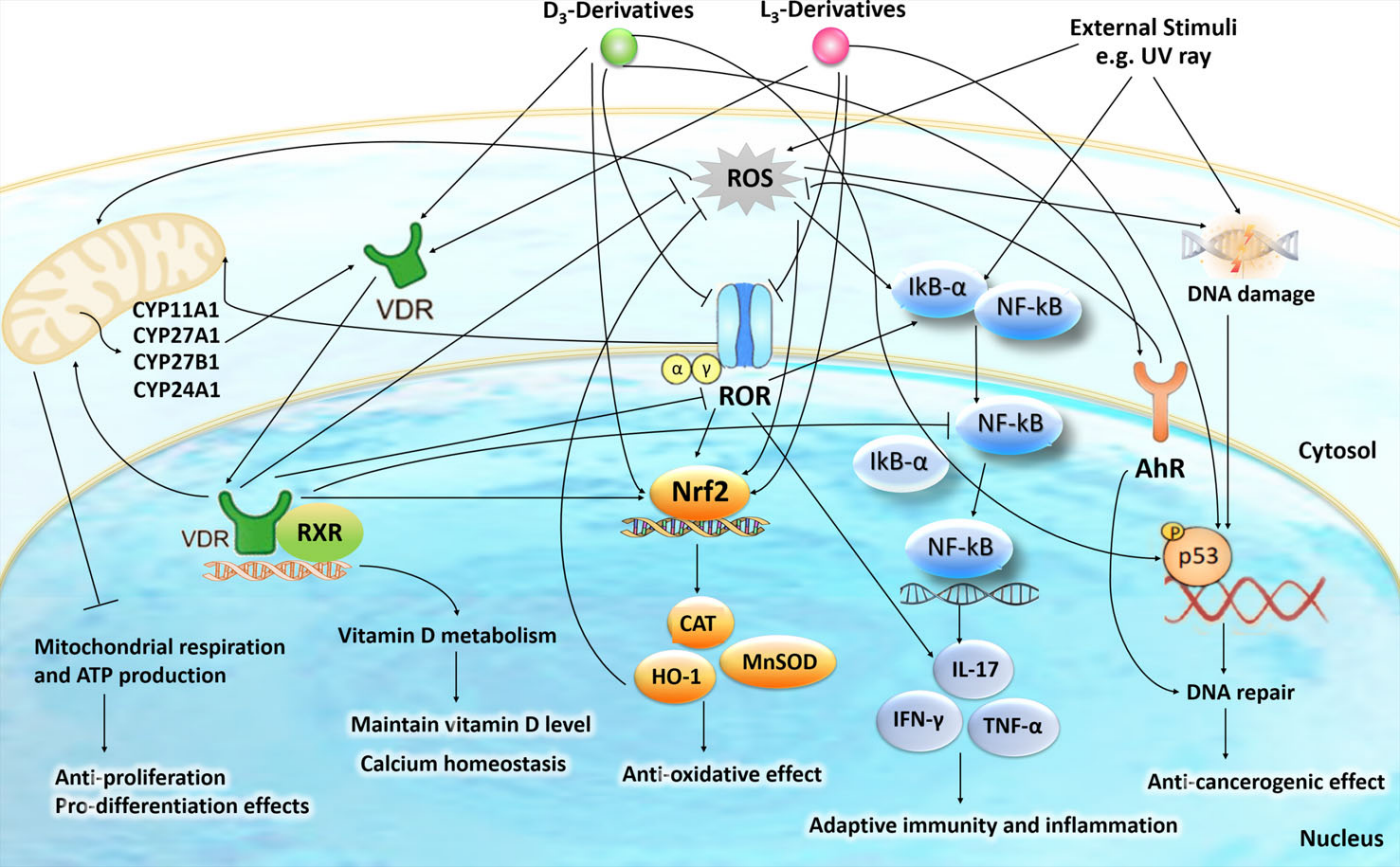
The intracellular action of vitamin D3 (D3)- and lumisterol (L3)-hydroxyderivatives in photoprotection against UVR. Signal transduction includes the activation of nuclear receptors such as vitamin D receptor (VDR), retinoic acid orphan receptor (ROR)α/γ, and aryl hydrocarbon receptor (AhR) and the direct action of D3- and L3-hydroxyderivatives on mitochondrial processes. The nuclear receptors activities are linked with the transcriptional master regulators NRF2 (nuclear factor erythroid-derived 2-like 2), p53 and NFκB (nuclear factor kappa-light-chain-enhancer of activated B cells) to coordinate anti-oxidative, DNA repair, anti-inflammatory, and antiproliferative as well as anti-carcinogenesis mechanisms. The figure is reprinted from (45) with a permission from the publisher.

**Figure 2:**
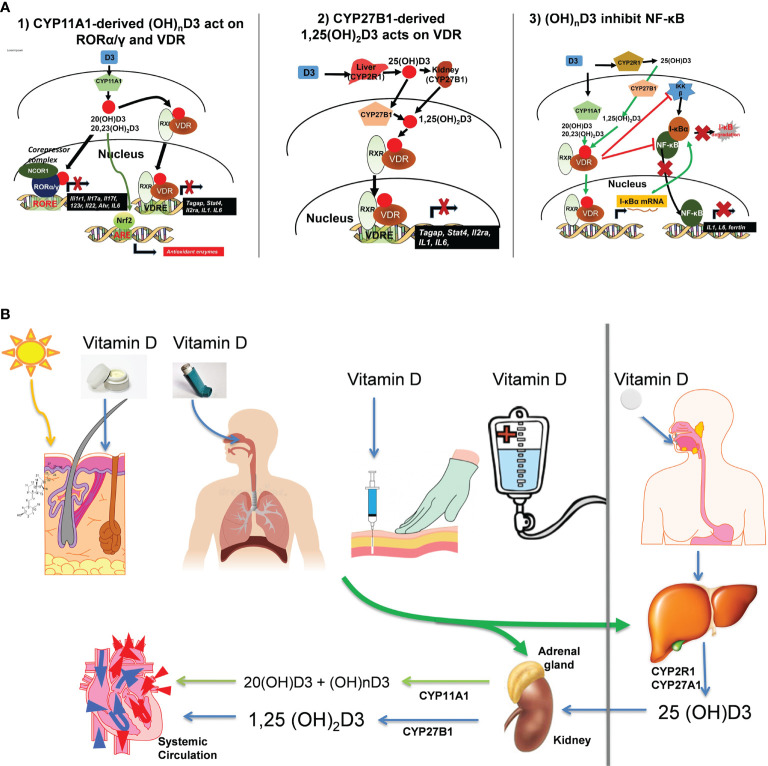
**(A)** Mechanism of action of canonical and non-canonical vitamin D-hydroxyderivatives. Vitamin D signaling in mononuclear cells downregulates inflammatory genes and suppresses oxidative stress. VDR, vitamin D receptor; RXR, retinoid X receptor; ROR, retinoic acid orphan receptor, ROR, ROR response element; ARE, antioxidant response element; VDRE, vitamin D response element; NRF2, nuclear factor erythroid-derived 2-like 2. **(B)** Different routes of vitamin D delivery will impact vitamin D activation pattern. The figure is reprinted from (46) with a permission from the publisher.

In the canonical pathway of the activation of vitamin D to 1,25(OH)_2_D3 involves sequential hydroxylations at C25 by CYP2R1 and CYP27A1 and at C1α by CYP27B1 occurring, respectively, in the liver and kidney (22, 23) and in peripheral organs including skin (24). In alternative pathway (non-canonical) vitamin D is activated by CYP11A1 through sequential hydroxylations of it side chain with additional metabolism by other CYP enzymes (23, 25–28). In addition, CYP11A1 is expressed in immune cells, raising a possibility that CYP11A1-derived vitamin D metabolites can be produced in immune cells to regulate their function in a cell autonomous manner (29). While 1,25(OH)2D3 exerts its phenotypic activity through activation of the VDR (30–35) and to some degree through non-genomic action on 1,25D3-MARRS receptor (36, 37), the CYP11A1-derived vitamin D metabolites, in addition on acting on the VDR (13, 38–41), can also interact with alternative nuclear receptors including retinoic acid receptors (RORs) (41, 42), aryl hydrocarbon receptor (AhR) (43) and liver X receptors (LXR) (44). It should be noted that 1,25(OH)2D3 can also act as an agonist on the AhR and LXRs (see **Figures 1** and **2** for details).

## VDR in the Crosstalk Between Keratinocytes and Melanocytes in Melanomagenesis

Under normal physiological conditions, melanocyte homeostasis is maintained by paracrine, autocrine, and direct cell-cell communication between melanocytes and adjacent keratinocytes that comprise epidermal melanin units (47, 48). During melanomagenesis, melanocytes begin to downregulate expression of adhesion molecules, such as E-cadherin, enabling an epithelial-mesenchymal transition that severs transforming melanoma cells from the regulatory activity of adjacent keratinocytes. This process then enables the tumor to take control of its epidermal microenvironment (49). It is known that Wnt/β-catenin signaling is a key regulator of melanocyte-keratinocyte adhesion and interactions; however, the exact role it plays is complicated. Some studies indicate activation of Wnt/β-catenin signaling is associated with decreased melanoma cell proliferation and that loss of this signaling pathway might induce melanomegenesis (50). Indeed, Wnt/β-catenin signaling is important for melanocyte differentiation *via* activation of MITF expression and posttranslational processing (51). On the other hand, others have shown that Wnt/β-catenin signaling is essential for metastatic melanoma cell survival and its inhibition leads to reduced proliferation, migration, and invasion (52). These differing observations could result from differing influences of canonical (β-catenin dependent) or non-canonical Wnt signaling on melanomas during disease progression (53). Of note, active forms of vitamin D inhibit Wnt/β-catenin signaling in squamous cell carcinoma (54). Recently, there is evidence that points towards an inverse relationship with VDR expression and Wnt/β-catenin signaling in primary melanomas which yields reduced proliferation and immune response evasion (11). It could be that differences in VDR expression contribute to how Wnt/β-catenin signaling influences melanomas. The complex changes in vitamin D signaling and their roles in melanoma development, progression, and therapy have been also discussed recently (12, 55).

Also important in the crosstalk within the epidermal melanin unit is that VDR heterodimerizes with other nuclear receptors including retinoid X receptors (RXRs). We have previously shown that in a VDR null (VDR^-/-^) mouse model topically treated with the carcinogen 12-dimethyl-benz[a]anthracene (DMBA)-12-O-tetradecanoylphorbol-13 acetate (TPA) resulted in numerous melanocytic growths. In that same study, a separate mouse model harboring a conditional tissue-specific keratin 14 promoter-driven cre-mediated epidermal RXRα knockout (*RXRα^ep-/-^* mice) also exhibited melanocytic growths (10). These data indicated that both absence of VDR and keratinocytic RXRα knockout stimulated melanocytic growth following tumor promoting treatment. This observation was further explored in additional mouse models in which keratinocytic RXRα knockout was combined with two melanomagenic mutational backgrounds (*RXRα^ep-/-^|CDk4^R24C/R24C^* and *RXRα^ep-/-^|Tyr-NRAS^Q61K^*) and exposed to acute neonatal UVB irradiation in combination with adult chronic UVB doses. These mice exhibited increased melanocytic growth as had been seen previously. They also had elevated malignant melanocytic tumors and increased metastasis to the draining lymph nodes concurrent with a loss in skin expression of PTEN and P53 tumor suppressors (29). To further explore the contribution of keratinocytic RXRα towards melanomagenesis we generated a mouse model that combined the previous background mutations to generate a highly conducive mutational landscape (*RXRα^ep-/-^|Tyr-NRAS^Q61K^|CDk4^R24C/R24C^*). With this mouse model we observed the formation of spontaneous melanomas in the absence of UVB when keratinocytic RXRα was ablated. Following acute neonatal UVB irradiation, melanomas in adult keratinocytic RXRα ablated mice had increased radial and vertical growth phases, increased proliferation, increased angiogenesis, reduced apoptosis, and increased metastasis to the draining lymph nodes. We also noted in the tumor adjacent normal skin irradiated with UVB that there was increased expression of activated AKT, p21, and cyclin D1 with reduced expression of pro-apoptotic marker BAX (30).

A significantly higher percent of cells from benign human nevi samples exhibited nuclear localization and strong expression of RXRα (P < 0.0001) compared to melanomas (with or without metastasis) (31). In the same report, primary human melanoma samples exhibited significantly higher cytoplasmic expression of RXRα compared to the nevi (P = 0.018) or the melanomas with metastasis and in metastasis samples (P = 0.004). The nuclear *vs* cytoplasmic expression of transcription factor such as RXRα could be critical for regulating their target gene expression by limiting its interaction with their heterodimeric partners and cytoplasmic localization could be essential to mediate the non-genomic actions of RXRα. A previous study by Boehm et al. also showed decreased expression of RXRα in human melanocytic tumors (32). Above results argue for its anticancerogenic role and suggest a cell-autonomous role of melanocytic RXRα in melanoma suppression.

Interestingly, strong nuclear expression of RXRα is also reported in epidermal keratinocytes of normal human skin and we reported for the first time that its expression is reduced or lost in skin keratinocytes adjacent to melanocytic tumors during melanoma progression in humans (33) suggesting a non-cell autonomous role of keratinocytic RXRα in suppressing melanoma progression. In contrast, cytoplasmic intensity of RXRβ did not differ significantly between groups of nevi and melanoma. Although, cytoplasmic expression of RXRβ was significantly reduced in human metastasis samples compared to the human melanoma samples (31) indicating a role of RXRβ in mediating melanoma metastasis.

We have also shown that both VDR and keratinocytic RXRα contribute towards photoprotection of melanocytes against UVB radiation *in vivo* using preclinical studies in mice models. Using the *RXRα^ep-/-^* mouse model subjected to acute neonatal UVB irradiation, we demonstrated that the absence of keratinocytic RXRα resulted in increased DNA damage, proliferation, and migration of melanocytes *in vivo*. We then confirmed these results *ex vivo* using primary melanocytes which exhibited increased growth in conditioned media generated from culturing isolated RXRα knockout keratinocytes. This was explained by increased expression of keratinocyte secreted growth factors ET-1, FGF2, and SCF in the skin of *RXRα^ep-/-^ mice* following UVB irradiation (34) underscoring a “non-cell autonomous” role of keratinocytic RXRα in UV-induced melanocyte homeostasis.

Interestingly, mice with melanocyte-specific ablation of RXRα and RXRβ (RXRα^mel-/-^ | RXRβ^mel-/-^) attract a reduced number of IFN-γ secreting immune cells than in wild-type mice following acute UVR, *via* altered expression of chemoattractive and chemorepulsive chemokines/cytokines. Reduced IFN-γ in the microenvironment modifies UVR-induced apoptosis, and due to this, the survival of dermal fibroblasts is significantly decreased in mice lacking RXRα/β (35). Results demonstrate that melanocytic-RXRs in a “non-cell autonomous” manner modulate post-UVR survival of dermal fibroblasts highlighting a role in immune surveillance, while independently in a “cell autonomous” manner regulate post-UVR melanocyte survival (35).

We have also demonstrated that melanocytic VDR also affords photoprotective properties in a different mouse model in which melanocytic VDR was ablated (*VDR^mel-/-^*). When knockout mice were subjected to acute neonatal UVB irradiation they exhibited fewer differentiated melanocytes with reduced proliferation, reduced apoptosis, and increased DNA damage (36).

Interestingly active forms of vitamin D3 show photoprotective activities in both melanocytes and keratinocytes (37–43) through various mechanisms also including the VDR (40, 44, 56, 57).

Altogether, above data highlight the importance of nuclear receptor signaling in melanocytes driven by VDR and its principal heterodimer partners RXRα and RXRβ in the regulation of melanocyte homeostasis and melanomagenesis in the skin and tumor microenvironment. Our data further underscores a non-cell autonomous role of RXRα both in keratinocytes and melanocytes of the skin in controlling melanocyte homeostasis and melanomagenesis.

## Vitamin D Receptor Polymorphisms in Melanoma

The VDR gene is located on chromosome 12q13.11 and has 11 exons (58). Over 600 single nucleotide polymorphisms have been identified in the VDR gene including FokI (C/T-rs2228570, previously named rs10735810), TaqI (rs731236), BsmI (rs1544410), and ApaI (rs7975232) which are the most commonly analyzed in relation to melanoma (5). Cdx2 (rs11568820), EcoRV (rs4516035), BglI (rs739837) have also been studied in this context, but to a lesser extent.

The FokI polymorphism (C/T-rs2228570, previously named rs10735810) is located on exon 2 of the VDR gene (5). This polymorphism creates a new start codon 10 base pairs upstream from the usual start codon, leading to a longer VDR protein that is less active compared to the shorter protein variant. The shorter protein variant is 424 amino acids and corresponds to the C nucleotide allele or F allele, and the longer 427 amino acid variant corresponds to the F allele (59, 60). The TaqI polymorphism (rs731236) is located at codon 352 of exon 9 of the VDR gene, and functions as a restriction fragment length polymorphism (5). It creates a silent codon change of ATT to ATC, which both code for isoleucine (5, 61).

The BsmI polymorphism (rs1544410) also acts as a restriction fragment length polymorphism that results in a silent mutation (5, 61). It is located in intron 8 at the 3^rd^ end of the VDR gene, thus it may affect VDR gene expression and mRNA stability (60). The ApaI polymorphism is located near the BsmI polymorphism, and thus, may have similar effects (5, 61). The Cdx2 (rs11568820) polymorphism is located in the promoter region of the VDR gene, and results in an adenine replacing a guanine (5, 61). The EcoRV polymorphism (rs4516035) is also located in the promoter region of the VDR gene, and is thought to play a role in the anticancer immune response (5, 62). Lastly, the BglI polymorphism (rs739837) is located near the stop codon in exon 9 (5).

A 2020 meta-analysis calculated the odds ratios and 95% confidence intervals for the dominant and recessive models for 7 VDR gene polymorphisms (63). The dominant model (Bb + BB *vs*. bb) of Bsml (rs1544410) showed a statistically significant 15% risk reduction in malignant melanoma incidence for carriers of the rarer allele B. Carriers of the rarer allele f (Ff + ff *vs*. FF) of FokI (rs2228570) were shown to be 22% more likely to develop malignant melanoma. Additionally, for ApaI (rs7975232), there is a 20% higher risk of melanoma for carriers of the rarer a allele (Aa + aa *vs*. AA). No significant association between melanoma risk and the other investigated VDR polymorphisms, which included TaqI (rs731236), A-1012G (rs4516035), Cdx2 (rs11568820), and BglI (rs739837), was found.

## VDR Expression as a Prognostic Biomarker

One cohort-study assessed the relationship between VDR expression and prognostic factors in Central European cohort of melanoma patients (64, 65). VDR expression was quantified immunohistochemically in 69 cutaneous melanomas and compared to the tumors’ pTNM (pathological tumor, node, metastasis) stage, ulceration, and tumor-infiltrating lymphocytes. pTNM staging is based on the tumor (i.e., Breslow thickness, ulceration), spread to nearby lymph nodes, and distant metastases. The higher the tumor’s stage, the worse the prognosis.

Strongest and highest VDR expression was detected in the nuclei of epidermal keratinocytes for normal uninvolved skin compared to melanocytic lesions. For “nuclear localization”, VDR expression decreased in the following order: *normal skin > melanocytic nevi > primary melanomas = metastases* (64). For “cytoplasmic localization”, VDR expression decreased in the order: *normal skin = melanocytic nevi > primary melanomas = metastases*. Reduction in VDR expression with the development of the pigmented lesions was more evident in the nuclei than in the cell-cytoplasm suggesting a cell-autonomous role of canonical VDR signaling in the melanocytes during melanoma progression and metastasis (64).

Interestingly, VDR expression in the basal and supra-basal keratinocytes of the skin epidermis surrounding the melanocytic tumors was markedly lower in comparison to normal skin without any skin lesions, which also suggests a non-cell autonomous role of keratinocytic VDR in melanomagenesis (64). Further, high VDR expression both in primary and metastatic melanomas was a factor that favorably influenced the OS in melanoma cohort.

In melanoma, ulceration contributes to the tumor of pTNM staging, and is a hallmark of more aggressive tumors. Whereas, the presence of tumor-infiltrating lymphocytes in melanoma is associated with a favorable prognosis. Less advanced melanomas, like those with fewer than three lymph node metastases and those without distant metastases, had the strongest VDR expression (64, 65). Whereas tumors with indicators of poor prognosis like ulceration or non-brisk or absence of tumor-infiltrating lymphocytes, showed significantly lower VDR expression. Most importantly patients with metastatic disease and *VDR^-/-^* melanomas had the poorest probability of survival (64, 65). Interestingly, the expression of activating vitamin D enzyme CYP27B1 was inversely correlated with melanoma progression and overall and disease-free survival times and such correlation was amplified by a concomitant decrease in the VDR expression (55, 65, 66). While CYP24A1 levels were high in nevi and early-stage melanomas in comparison to normal epidermis, its level decreased during melanoma progression similarly to CYP27B1 and VDR (67). These findings indicate that vitamin D signaling system including VDR expression plays an important role in melanoma prognosis and may also be used as an additional prognostic biomarker. Similar trend was reported for ocular melanoma (68). Importantly, recent experimental studies have shown that knocking out of the VDR in melanoma cells increase their malignant behavior and decreases responsiveness to active form of vitamin D indicating that the VDR can serve as the melanoma tumor suppressor gene (69), which is consistent with the role of the VDR as the tumor suppressor gene in the skin as originally proposed by Bikle (44). Of note, defects in VDR lead to increased malignant behavior in other tumors including bladder, ovarian, lung and breast cancers, lymphomas (70–75).

There was a reverse correlation between melanin content and expression of the VDR and CYP27B1 as well as of RORα and γ in human melanoma samples (64, 66, 76). RORα and γ, alternative receptors for vitamin D-hydroxyderivatives, are expressed at lower levels in melanomas than in nevi and their expression decreases during melanoma progression, with lowest expression found in stage III and IV melanomas and in metastases (76). Interestingly, the expression of VDR as wells as of RORs was related to the HIF1α activity, which also affected FoxP3 expression in metastatic melanoma (77). Of note, melanogenesis can stimulate HIF1α expression and anaerobic glycolysis in melanoma cells (78) explaining in part the correlation between defects in VDR expression and signaling and defective responses to vitamin D in pigmented melanoma cells (64, 79, 80).

A separate study conducted by Muralidhar et al. analyzed 703 primary melanoma transcriptomes to better understand the role of vitamin D-VDR signaling (11). They found that VDR expression was independently protective against melanoma-related death in both primary and metastatic disease. VDR expression was shown to be inversely related to Wnt/β-catenin signaling, suggesting a mechanism for the anti-proliferative effects of vitamin D-VDR signaling. Additionally, increased VDR expression was associated with the upregulation of pathways involving the antitumor immune response as demonstrated by a greater abundance of tumor-infiltrating lymphocytes. This study further supports VDR’s utility as a prognostic biomarker, especially in those patients considering immunotherapy. It also establishes a causal relationship between vitamin D-VDR signaling and melanoma survival, suggesting that this mechanism could serve as a target for pharmacologic agents.

In addition to its generalized expression, the subcellular localization of VDR to the nucleus also could be beneficial as a biomarker for melanoma progression. Hutchinson et al. studied 34 benign nevi, 149 metastatic melanomas, and 44 matched metastases *via* immunohistology for the subcellular localization of VDR and phosphorylated ERK (p-ERK) as an indicator of MAPK activation (81). They found that as melanomas progressed, they exhibited reduced nuclear localization of VDR and increased cytoplasmic localization. Overall, expression of VDR decreased from benign nevi to metastatic melanoma and further decreased in metastasizing primary tumors. When they observed VDR localization in malignant melanomas known to have metastasized and compared them to those known to not have metastasized within five years, they saw nuclear VDR was reduced while there was no difference in cytoplasmic localization. They also found increased p-ERK consistent with cytoplasmic localization of VDR likely a result of the known mechanism of MAPK inhibition of VDR signaling when it is heterodimerized to RXRα *via* phosphorylation of serine 260 (82). These observations highlight the need for more research on the usefulness of VDR nuclear localization as a prognosticator for metastasizing melanomas.

## Serum Vitamin D Levels and Prognosis

As part of the Leeds Melanoma Cohort, Newton-Bishop et al. reported an association between higher 25-hydroxyvitamin D3 serum levels at time of melanoma diagnosis and lower Breslow thickness (p value= .002) (83). Higher 25-hydroxyvitamin D3 levels were also found to be associated with increased survival independent of Breslow thickness. Several other studies have confirmed an association between higher serum vitamin D levels at diagnosis and better prognosis in melanoma (84–86). However, a more recent study asserts that rather than high levels of vitamin D being protective a deficiency in vitamin D (<25 nmol/L) actually shortens patient survival time from melanoma in a VDR-dependent manner (11).

Additionally, an observational single center study with estimated study completion date of January 2021, not yet published, is investigating the response to treatment with anti-programmed death 1 (PD-1) therapy in relation to serum vitamin D levels in 40 advanced melanoma patients (ClinicalTrials.gov Identifier: NCT03197636) (87). Serum levels of vitamin D will be measured at baseline, 3, and 6 weeks after initiation of treatment with anti-PD1 therapy followed by three years of observational follow-up. Response to treatment will be assessed at each visit within the study period and at follow-up.

## Vitamin D, VDR and Immunotherapy

The issue of interference of active forms of vitamin D on immunotherapy deserves special attention, especially that immunotherapy represents the promising therapeutic approach against melanoma (88–95). In this context, inhibitory role of vitamin D in the adaptive immune responses (96, 97) requires explanation. Although it inhibits T cell responses in autoimmune responses (98), the evidence that it acts as an immunosuppressor is missing. On the opposite, it is inhibiting proinflammatory responses through VDR mediated inhibition of NFκβ and inverse agonism on RORγ and inhibition of oxidative stress through activation of NRF2-dependent pathways (45, 46, 57, 99). However, it is unclear to which degree, how, and whether it will inhibit anti-tumor T-cell responses. On the other hand, vitamin D activates the innate immune system (96, 97), which plays an important role in anti-tumor activity (100–106). Therefore, the actions of active forms of vitamin D can be defined as immunoregulatory, with their full definitions requiring future careful studies.

## Vitamin D and Its Analogs in the Treatment of Melanoma

Several studies are investigating the use of vitamin D or its analogs as adjuvant treatment in melanoma patients with an understanding that different delivery routes will influence vit D activation (**Figures 2B** and see below).

One report that utilized data from the Women’s Health initiative (WHI) calcium/vitamin D randomized controlled trial, studied the effects of calcium and low-dose vitamin D on the risk of non-melanoma and melanoma skin cancers in post-menopausal women (107). Women ages 50-79 years (N=36,282) were randomly assigned to receive 1,000 mg of elemental calcium plus 400 IU of vitamin D3 daily or placebo for a mean follow-up period of seven years. Non-melanoma and melanoma skin cancer diagnoses were self-reported annually. The study concluded that the treatment group and control group showed no significant difference in the incidence of melanoma or non-melanoma skin cancers. However, women on the calcium/vitamin D regiment with a history of non-melanoma skin cancer had a reduced risk of melanoma as opposed to those receiving placebo (hazard ratio 0.43; 95% confidence interval: 0.21 to 0.90: P(interaction) =.038). It was also noted that this difference was not seen in women that did not have a history of non-melanoma skin cancer.

In 2010, the Australia and New Zealand Melanoma Trials Group conducted a pilot randomized placebo-controlled phase II trial, Mel-D, to investigate the safety and efficacy of adjuvant high-dose vitamin D administration in patients with cutaneous melanoma that had initially been treated with wide excision (Australian New Zealand Clinical Trials Registry #ACTRN12609000351213) (108, 109). The adjuvant treatment included an oral loading dose of 500,000 IU Vitamin D followed by a once monthly oral dose of 50,000 IU Vitamin D for two years. Patients in this study reportedly experienced an improvement in progression-free survival and overall survival.

The ongoing study, VidMe, is a multicenter randomized placebo-controlled phase III trial intended to examine the efficacy and long-term safety of high-dose vitamin D supplementation in 500 patients with melanoma (ClinicalTrials.gov Identifier: NCT01748448) (110, 111). Once a month, participants will either receive 100,000 IU of vitamin D or placebo (Arachidis oleum raffinatum). This study’s primary endpoint is relapse-free survival. They also plan to assess the expression of VDR in the primary tumor and its possible correlation with relapse. Secondarily, vitamin D levels at diagnosis will be correlated with melanoma site, subtype, and stage at diagnosis. Vitamin D levels will continue to be monitored after supplementation to determine if serum levels depend on the genetic variability of the vitamin D pathway. Additionally, they plan to investigate whether VDR immunoreactivity correlates with stage at diagnosis.

Vitamin D analogs have also exhibited promising photoprotective and anticancer properties (13, 57, 112) indicating their possible application to counteracting skin cancer, including melanomas. The anti-melanoma activity of the non-calcemic analog, 20(OH)D3, was shown in a preclinical *in vivo* model (113). 20(OH)D3 is non-calcemic but possesses similar antiproliferative activity *in vitro* when compared to 1,25(OH)2D3. Skobowiat et al. demonstrated decreased colony formation both in the monolayer and soft agar conditions when cells were treated with 20(OH)D3. 20(OH)D3 was also shown to inhibit melanoma cells in transwell migration and spheroid toxicity. Additionally, 20(OH)D3 decreased melanoma tumor growth in immunocompromised mice without obvious signs of toxicity. These results suggest that 20(OH)D3 is likely effective and safe, and thus, should undergo further preclinical testing as an antimelanoma therapy.

Therefore, cellular expression of RXRs and VDR in addition to their sub-cellular localization could be used as a prognostic biomarker for melanoma progression in humans. While vitamin D3 and its analogs are currently being explored in pre-clinical and clinical settings as a possible adjuvant therapy in the treatment of melanoma (107, 108, 110, 111, 113), in those individuals with decreased or dysfunctional VDR and RXR expression, vitamin D supplementation is unlikely to be beneficial. Thus, there is a need for a novel therapy that increases and/or restores functional VDR and RXR expression in conjunction with the supplementation of vit D or its analogs. Similarly, the *in vivo* anti-melanoma effects of the novel vit D analogs need to be established and the underlying mechanisms of action need to be deciphered.

## Author Contributions

All authors have contributed intellectually for the preparation of the manuscript. All authors contributed to the article and approved the submitted version.

## Funding

Research reported in this publication was supported in part by National Institute of Environmental Health Sciences (NIEHS) of the National Institutes of Health (NIH) under the award number 1R01ES016629-01A1 (PI :AI), the OSU/OHSU College of Pharmacy Pilot Project Grant (PI :AI), NIH grants to ATS including 1R01AR073004-01A1, R01AR071189-01A1, and a VA merit grant [no. 1I01BX004293-01A1 (PI ATS)], and a Training grant from the National Center for Complementary and Integrative Health (NCCIH) of the National Institutes of Health under award number T32AT010131.

## Conflict of Interest

The authors declare that the research was conducted in the absence of any commercial or financial relationships that could be construed as a potential conflict of interest.

## Publisher’s Note

All claims expressed in this article are solely those of the authors and do not necessarily represent those of their affiliated organizations, or those of the publisher, the editors and the reviewers. Any product that may be evaluated in this article, or claim that may be made by its manufacturer, is not guaranteed or endorsed by the publisher.
